# Effect of Triton all-in-one irrigant on electronic working length determination using two apex locators: an *in vitro* study

**DOI:** 10.7717/peerj.20872

**Published:** 2026-02-18

**Authors:** Aybüke Karaca Sakallı, İsen Güleç Koçyiğit, Bilge Özcan

**Affiliations:** 1 Faculty of Dentistry, Department of Endodontics, Ankara Medipol University, Ankara, Turkey; 2 DentTarz Academy Dental Clinic, Ankara, Turkey

**Keywords:** Citric acid, Endodontics, Ethylenediaminetetraacetic acid, Root canal irrigants, Sodium hypochlorite, Triton

## Abstract

**Background:**

This study evaluated whether Triton, a newly introduced all-in-one irrigant, affects the accuracy of electronic working length determination, and compared two electronic apex locators (Ai-Pex, Propex Pixi) under different irrigant conditions.

**Materials and Methods:**

Forty-four extracted single-rooted human teeth were embedded in alginate. Actual working length was determined under a dental operating microscope. Specimens were assigned to four groups (Triton, 17% ethylenediaminetetraacetic acid, 10% citric acid, dry canal). Electronic working lengths were measured with Ai-Pex and Propex Pixi using #15 K-files. Statistical analyses were performed using one-way and two-way analysis of variance (ANOVA) following normality assessment, with statistical significance set at *p* < 0.05.

**Results:**

No significant differences occurred among Triton, ethylenediaminetetraacetic acid, and citric acid groups (*p* > 0.05). Dry canal measurements were significantly longer than the actual working length measured under a dental operating microscope (*p* < 0.05). Both apex locators showed comparable accuracy.

**Conclusions:**

Triton did not reduce apex locator precision, supporting its potential as a time-saving irrigant that permits simultaneous irrigation and measurement. Further clinical validation is required.

## Introduction

In endodontics, effective removal of microorganisms and prevention of reinfection rely on thorough cleaning and sealing of the root canal system. Achieving these outcomes requires meticulous preparation, disinfection, and obturation extending from the coronal access to the apical constriction (Kuttler, 1955, 1958). The apical constriction, also referred to as the minor apical foramen, serves as a critical anatomical landmark for optimal treatment success (Duran-Sindreu et al., 2013). Clinical and histological studies consistently show that treatment outcomes are more favorable when the procedure terminates precisely at this location, whereas over- or under-instrumentation compromises prognosis.

Various techniques are used to determine working length, including radiography, tactile feedback, and anatomical knowledge (Berman & Hargreaves, 2020). Among these, the combination of periapical radiographs and electronic apex locators (EAL) is the most commonly used method in modern endodontics (Cîmpean et al., 2023; Meares & Steiman, 2002).

In 1962, Sunada introduced the electrical resistance principle that forms the basis of modern electronic apex locators, and EAL technology has evolved substantially since then (Sunada, 1962). Early generations were highly sensitive to canal conditions and irrigants, limiting their clinical reliability (Rifaat et al., 2023). Third-generation devices introduced dual-frequency ratios (Cîmpean et al., 2023; Berman & Hargreaves, 2020), while fourth- and fifth-generation models incorporated advanced algorithms and multifrequency analysis to enhance precision (Berman & Hargreaves, 2020; Chukka et al., 2020). Although fourth-generation models provided theoretical advancements, studies reported no significant increase in accuracy compared to previous versions (Duran-Sindreu et al., 2013). The latest sixth-generation EALs further improved measurement consistency by accounting for the influence of various irrigants (Shekarbaghani, Bolhari & Khalilak, 2024).

Among these devices, Propex Pixi (Dentsply Maillefer, Ballaigues, Switzerland), a fifth-generation apex locator introduced in 2012, and the more recent Ai-Pex (Woodpecker Medical Instrument Co., Guilin, China; Guilin Woodpecker Medical Instrument Co. LTD, 2023; Çelebi Keskin & Yalçın, 2025), both use multifrequency impedance analysis. They are capable of reliable length determination in both dry and wet canals. These advances in EAL technology raise important questions regarding the influence of different irrigating solutions on measurement accuracy, particularly with newer combination irrigants.

Triton (Brasseler, Savannah, GA, USA) is a recently introduced all-in-one irrigant that combines sodium hypochlorite, citric acid, and cationic surfactants in a single formulation. It is designed to streamline irrigation protocols by offering antimicrobial activity, tissue dissolution, and smear layer removal in a single step (Sheng et al., 2023). Although its composition has demonstrated promising physicochemical and biological properties, such as biofilm inhibition and enhanced biocompatibility, its interaction with EALs remains unstudied.

Considering the significance of irrigant conductivity and pH on the performance of multifrequency EALs, it is crucial to understand how complex irrigants like Triton affect measurement accuracy for clinical use. So far, no research has explored the impact of Triton (Brasseler) on working length determination with EALs.

Therefore, this *in vitro* study aimed to assess the impact of three irrigants—Triton (Brasseler), 17% EDTA, and 10% citric acid—on the accuracy of two electronic apex locators, Ai-Pex (Woodpecker Medical Instrument Co.) and Propex Pixi (Dentsply Maillefer). Dry canal conditions were included as a control. By comparing a novel multi-agent irrigant with conventional solutions, this study addresses a key gap in the literature and provides new insights into the compatibility of contemporary irrigants with multifrequency EAL systems.

The null hypothesis was that there would be no significant difference in the accuracy of working length determination among the tested irrigants and EALs.

## Materials and Methods

This study was approved by the Ethics Committee of Ankara Medipol University (Approval No: E-85859696-604.01.01-1839). All procedures involving extracted human teeth were conducted per the ethical standards of the Declaration of Helsinki and its later amendments. Written informed consent for the use of extracted teeth was obtained from all donors before inclusion. The teeth were collected anonymously after routine extractions that were not performed specifically for research purposes, and no personally identifiable information was recorded.

### Sample size calculation

The sample size for this study was calculated using the G*Power analysis software. Based on the study by Yahya, Alchalabi & Alkhalidi (2024), with a confidence level of 95% and power of 80%, the minimum required sample size was determined to be 44 teeth, with 11 specimens in each group.

### Tooth selection and preparation

Forty-four freshly extracted, caries-free, single-rooted human maxillary central incisors with mature apices and straight canals (<10° curvature, measured according to Schneider’s method) were selected. The extracted maxillary central incisors were obtained from patients who required tooth extraction for orthodontic alignment or periodontal reasons. No teeth were extracted specifically for the purpose of this study. Teeth presenting root cracks, external or internal resorption, canal calcifications, immature apices, previous endodontic treatment, coronal restorations, or root fractures were excluded. All specimens were examined under a dental operating microscope (Leica M320; Leica Microsystems, Wetzlar, Germany) at ×25 magnification to confirm eligibility.

After extraction, the teeth were stored in 0.5% chloramine-T (Merck, Germany) for 48 h for disinfection, and calculus and soft tissue remnants were removed ultrasonically. Thereafter, the teeth were transferred to 0.1% thymol solution and stored at 4 °C until use. Prior to experimentation, the specimens were rinsed thoroughly with distilled water and kept in 0.9% sterile saline to prevent dehydration.

### Access cavity preparation and working length determination

**Figure 1:**
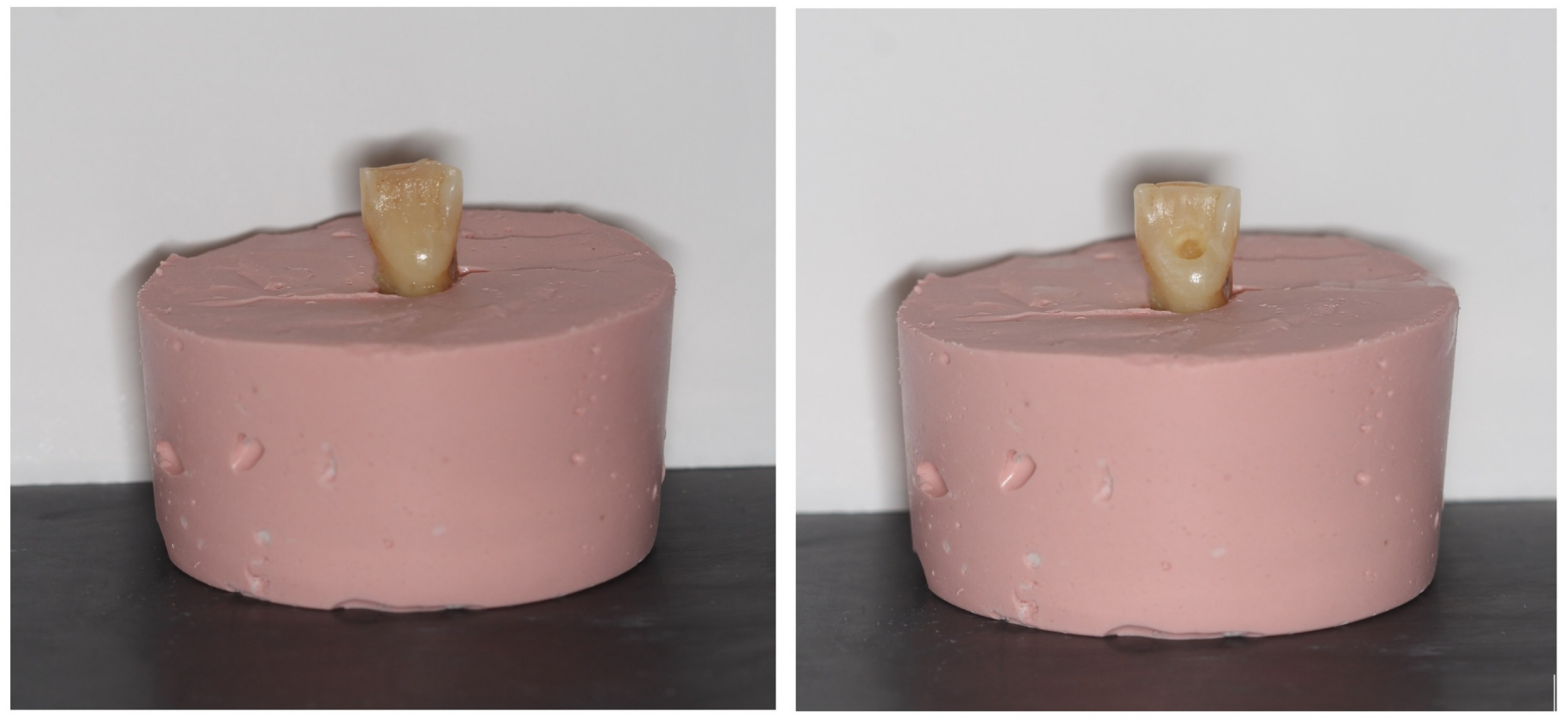
Standardized access cavity preparation and flattening of the incisal edge. (A) Tooth before access cavity preparation and flattening of the incisal edge. (B) Tooth after standardized access cavity preparation and flattening of the incisal edge.

After disinfection and storage, each tooth received a standardized access cavity preparation under a dental operating microscope. A standardized access cavity was prepared for all samples (Fig. 1). The incisal surfaces were flattened using a diamond disc to establish a consistent reference point, and standardized access cavities were prepared with a round diamond bur. Each tooth was numbered from 1 to 44 and randomly allocated to one of the four experimental groups by sequential assignment following random mixing of the samples, ensuring an equal distribution among groups.

**Figure 2:**
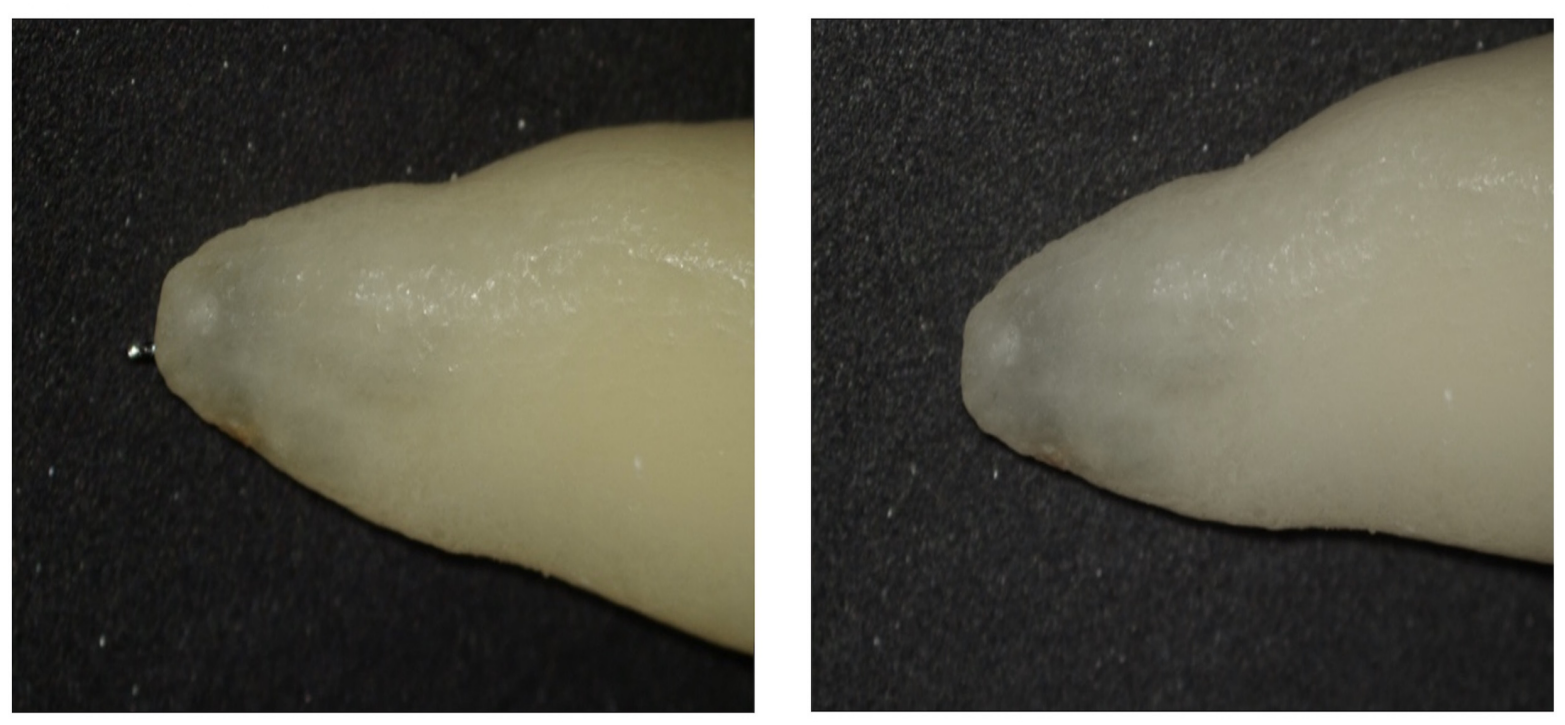
AWL measurement under dental operating microscope. Experimental setup used for electronic working length measurements. Extracted teeth were embedded in alginate to simulate periapical tissues, and measurements were performed using Ai-Pex and Propex Pixi electronic apex locators under different irrigant conditions.

The actual working length (AWL) of each canal was determined under a dental operating microscope (Leica M320, Leica Microsystems, Wetzlar, Germany) at ×25 magnification. A size 15 K-file (Dentsply Maillefer, Ballaigues, Switzerland) was gently advanced into the canal until the file tip was just visible at the major apical foramen (Fig. 2). The distance from the coronal reference point to the file tip was measured with a digital caliper (accuracy 0.01 mm) and recorded as the reference length. For standardization, the AWL was defined as 0.5 mm short of the major apical foramen, and tolerance margins of ±0.5 mm and ±1.0 mm from the reference length were used to evaluate the accuracy of electronic working length (EWL) measurements.

All measurements were independently performed and repeated three times by two calibrated observers who were blinded to the irrigation groups. The mean values of the measurements were recorded. Interobserver reliability was assessed using the intraclass correlation coefficient (ICC). In cases of disagreement between observers, the measurements were jointly re-evaluated under a dental operating microscope until a consensus was reached.

### Experimental setup

To simulate the conductivity of periapical tissues, each root was embedded in an alginate model (Phase, Zhermack SpA, Badia Polesine, Italy), leaving the apical 2 mm exposed. Lip clips were inserted into the alginate for EAL measurements. All measurements were completed within 2 h of model preparation to maintain alginate moisture. Four groups were designed based on the irrigation protocols: (Table 1).

**Table 1:** Irrigant solutions used in the study and their compositions. Composition, manufacturer, and country of origin for the irrigants tested: Triton all-in-one irrigant, 17% EDTA, and 10% citric acid. Triton is a dual-part solution combining sodium hypochlorite with chelating and surfactant agents.

Solution	Manufacturer (Country)	Composition
Triton All-in-One	Brasseler (USA)	Part A:1, 2, 4 Butanetricarboxylic acid, 2-phosphono, citric acid, sodium dodecylbenzenesulfonate, alcohols (C9-11, ethoxylated), polyethylene glycol 4-(tert-octylphenyl) ether, liquid, sodium lauryl sulfate, 2-ethylhexyl sodium sulfate, sodium cumenesulphonate, sodium hydroxide Part B: Sodium hypochlorite, sodium hydroxide
EDTA	Cerkamed, Stalowa Wola, Poland	17% EDTA solution
Citric Acid	Cerkamed, Stalowa Wola, Poland	40% Citric Acid solution

Group 1: Triton (Brasseler)

Group 2: 17% EDTA (Cerkamed)

Group 3: 10% citric acid (prepared from 40% Cerkamed solution *via* dilution)

Group 4: Dry canal (no irrigant)

Before working length determination with a size 15 K-file, pulpal tissue was removed from the root canals using a barbed broach (tirnef), followed by irrigation until the canals were visually confirmed to be clean. All canals were initially irrigated with 1 mL of sterile saline and dried with paper points. Subsequently, the canals were irrigated with 2 mL of the group specific solution delivered through a 27-gauge side-vented irrigation needle (Ultradent Endo-Eze, South Jordan, UT, USA). Excess solution in the pulp chamber was removed with air to maintain a moist but not flooded environment. Electronic working length (EWL) was determined using: Ai-Pex (Woodpecker Medical Instrument Co., Guilin, China), Propex Pixi (Dentsply Maillefer, Ballaigues, Switzerland) (Table 2).

**Table 2:** Electronic apex locators used in the study. Device name, manufacturer, and generation of the two electronic apex locators tested: Propex Pixi (5th generation) and Ai-Pex (new generation).

Device	Manufacturer	Description
Propex Pixi	Dentsply-Maillefer, Ballaigues, Switzerland	5^th^ generation apex locator
Ai-Pex	Woodpecker Medical Instrument Co., Guilin, China	7^th^ generation apex locator

**Figure 3:**
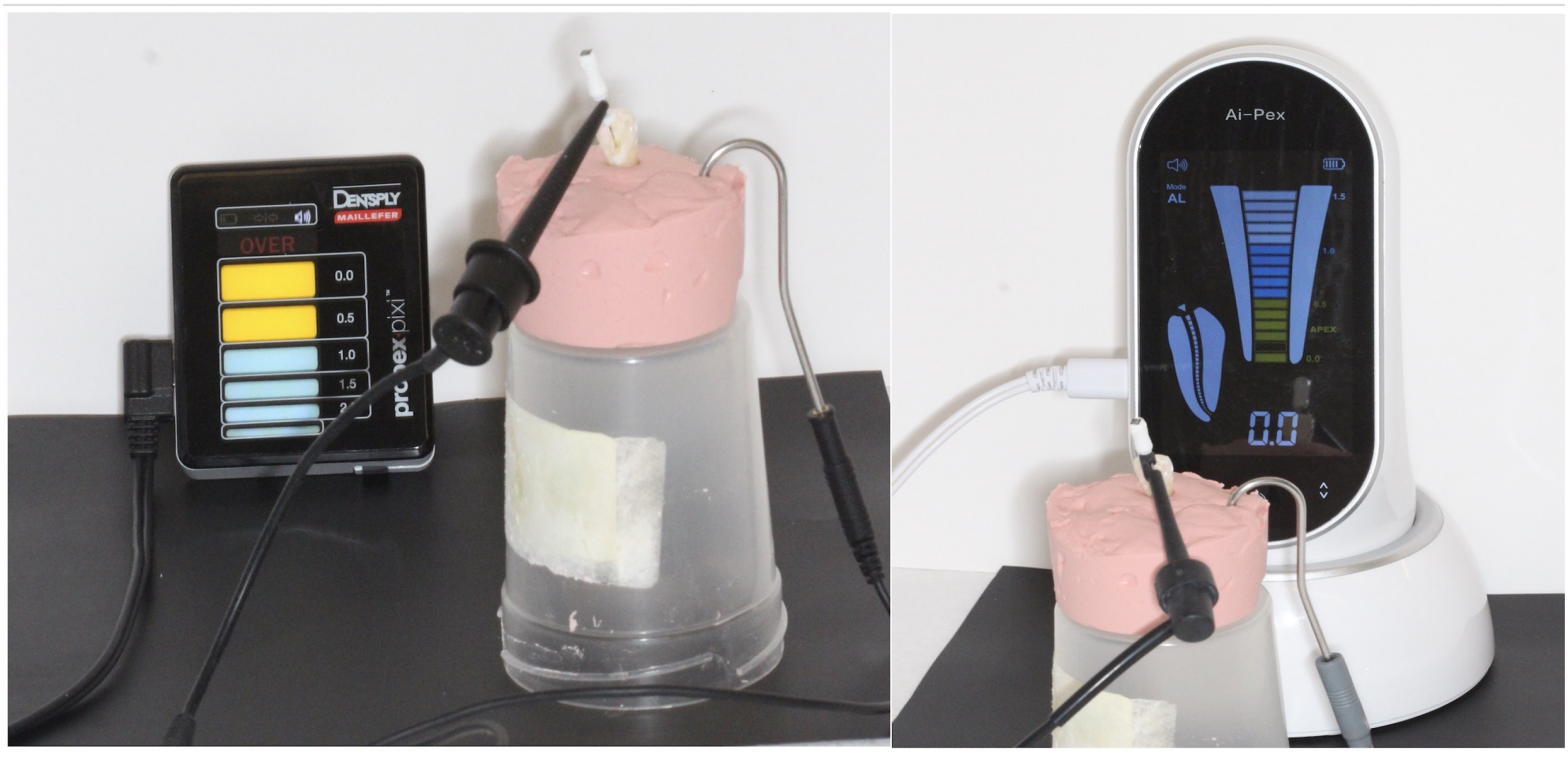
EWL determination using Ai-Pex and Propex Pixi in alginate model. Representative image of the electronic working length determination process using the Ai-Pex device. The file was advanced to the 0.0 mark on the device display, stabilized for 5 s, and retracted according to the manufacturer’s instructions.

For each device, a #15 K-file was introduced into the canal until the display reached and stabilized at the 0.0 mark for 5 s (Fig. 3). The file was then retracted according to the manufacturer’s instructions (Ai-Pex: 0.5–1 mm from the 0.0 mark, Propex Pixi: 0.5 mm from the 0.0 mark). The stopper was fixed with light-cured resin, and the file length was measured with a digital caliper (accuracy: 0.001 mm). Measurements were repeated three times and averaged. In Group 4, canals were dried thoroughly before measurement, and no irrigants were used.

### Statistical analysis

Normality was assessed using the Shapiro-Wilk test. Two-way analysis of variance (ANOVA) and *post-hoc* LSD tests with Bonferroni correction were used to evaluate differences in working lengths (α = 0.05). One-way ANOVA was applied to analyze differences between irrigant groups. Interobserver reliability was assessed using a two-way mixed-effects model to calculate ICC. Statistical analysis was conducted using SPSS v20.0 (IBM Corp., Armonk, NY, USA). A *p*-value < 0.05 was considered statistically significant. Accuracy was calculated as the percentage of electronic working length measurements falling within ±0.5 mm and ±1.0 mm of the actual working length.

## Results

Inter- and intraobserver reliability was excellent, with all ICC values exceeding 0.90 (*p* < 0.001), confirming high consistency and repeatability of working length (WL) measurements (Tables 3–5).

**Table 3:** Interobserver reliability for working length measurements. Intraclass correlation coefficients (ICC) for interobserver agreement on actual working length measurements determined under a dental operating microscope. Both single and average measures are shown, with 95% confidence intervals and F-tests.

Type	ICC	95% CI (Lower–Upper)	F	df1	df2	*p*-value
Single measures	0.899	[0.666–0.974]	18.831	9	10	<0.001
Average measures	0.947	[0.799–0.987]	18.831	9	10	<0.001

**Note:**

One-way random effects model where people effects are random.

**Table 4:** Intraobserver reliability for working length determination–Observer 1. Intraclass correlation coefficients (ICC) for repeated measurements by Observer 1, showing single and average measures, 95% confidence intervals, and F-test results.

	Intraclass correlation^b^	95% confidence interval		F test with true value 0			
		Lower bound	Upper bound	Value	df1	df2	Sig
Single measures	0.966^a^	0.872	0.991	53.169	9	9	0.000
Average measures	0.983^c^	0.932	0.996	53.169	9	9	0.000

**Notes:**

Two-way mixed effects model where people effects are random and measures effects are fixed.

a The estimator is the same, whether the interaction effect is present or not.

b Type A intraclass correlation coefficients using an absolute agreement definition.

c This estimate is computed assuming the interaction effect is absent, because it is not estimable otherwise.

**Table 5:** Intraobserver reliability for working length determination–Observer 2. Intraclass correlation coefficients (ICC) for repeated measurements by Observer 2, showing single and average measures, 95% confidence intervals, and F-test results.

	Intraclass correlation^b^	95% confidence interval		F test with true value 0			
		Lower bound	Upper bound	Value	df1	df2	Sig
Single measures	0.995^a^	0.981	0.999	375.497	9	9	0.000
Average measures	0.998^c^	0.990	0.999	375.497	9	9	0.000

**Notes:**

Two-way mixed effects model where people effects are random and measures effects are fixed.

a The estimator is the same, whether the interaction effect is present or not.

b Type A intraclass correlation coefficients using an absolute agreement definition.

c This estimate is computed assuming the interaction effect is absent, because it is not estimable otherwise.

No significant differences were observed between the actual working length and measurements obtained with Propex Pixi or Ai-Pex in the Triton, EDTA, and citric acid groups (*p* > 0.05). In contrast, both devices significantly overestimated WL under dry canal conditions (*p* < 0.05).

Comparison of the two apex locators revealed no significant differences across irrigant groups (*p* < 0.05), with both devices performing consistently in moist canal conditions (Table 6). Multiple comparison analysis showed overlapping 95% confidence intervals between the irrigant groups and the microscopic reference, confirming the absence of significant differences (Table 7).

**Table 6:** Mean working length measurements according to irrigant solution and device. Mean working lengths (mm) ± SD measured by the microscope, Ai-Pex, and Propex Pixi under four irrigant conditions. Uppercase letters indicate significant differences between irrigants within columns; lowercase letters indicate no significant differences between devices within rows (*p* > 0.05).

Irrigation solution	Microscope (Mean ± SD)	Ai-Pex (Mean ± SD)	Propex Pixi (Mean ± SD)
Triton	19.87 ± 1.18^Aa^	19.24 ± 1.06^Aa^	19.23 ± 1.09^Aa^
EDTA	20.49 ± 2.40^Aa^	20.02 ± 2.53^Aa^	19.85 ± 2.51^Aa^
Citric acid	20.49 ± 2.40^Aa^	19.94 ± 2.51^Aa^	19.80 ± 2.48^Aa^
Dry canal	22.84 ± 1.82^Ba^	22.39 ± 1.74^Ba^	22.30 ± 1.73^Ba^

**Note:**

*Different uppercase letters within the same column indicate statistically significant differences between solutions (*p* < 0.05). Different lowercase letters within the same row indicate no significant difference between measurement methods (*p* > 0.05).

**Table 7:** 95% confidence intervals of observed means for each irrigant solution and measurement method. Differences were assessed using *post hoc* tests following two-way ANOVA. The mean difference is significant at the 0.05 level.

Irrigation solution		95% confidence interval	
		Lower bound	Upper bound
Triton	Microscope	18.950	20.780
	Ai-Pex	18.327	20.156
	Propex Pixi	18.314	20.143
EDTA	Microscope	19.575	21.405
	Ai-Pex	19.101	20.930
	Propex Pixi	18.940	20.769
Citric acid	Microscope	19.575	21.405
	Ai-Pex	19.024	20.853
	Propex Pixi	18.887	20.716
Dry canal	Microscope	21.548	24.134
	Ai-Pex	21.096	23.682
	Propex Pixi	21.002	23.588

ANOVA demonstrated that WL differences were primarily influenced by the irrigant type (*p* < 0.001), with no significant effect of the measurement method or interaction (Table 8).

**Table 8:** Two-way ANOVA results for irrigant type and measurement method. Tests of Between-Subjects Effects from the two-way ANOVA model assessing the influence of irrigant solution, measurement method (device), and their interaction on working length differences. Partial Eta^2^ and observed power are reported.

Source	Type III sum of squares	Df	Mean square	F	Sig.	Partial Eta^2^	Observed power^b^
Corrected model	211.370^a^	11	19.215	4.467	0.000	0.199	1.000
Intercept	80,983.470	1	80,983.470	18,827.100	0.000	0.990	1.000
Irrigation solution	194.693	3	64.898	15.087	0.000	0.186	1.000
Measurement	14.484	2	7.242	1.684	0.188	0.017	0.352
Irrigation solution * Measurement	0.231	6	0.039	0.009	1.000	0.000	0.052
Error	851.683	198	4.301				
Total	87,226.811	210					
Corrected total	1,063.053	209					

**Notes:**

a R Squared = 0.199 (Adjusted R Squared = 0.154).

b Computed using alpha = 0.05.

**Figure 4:**
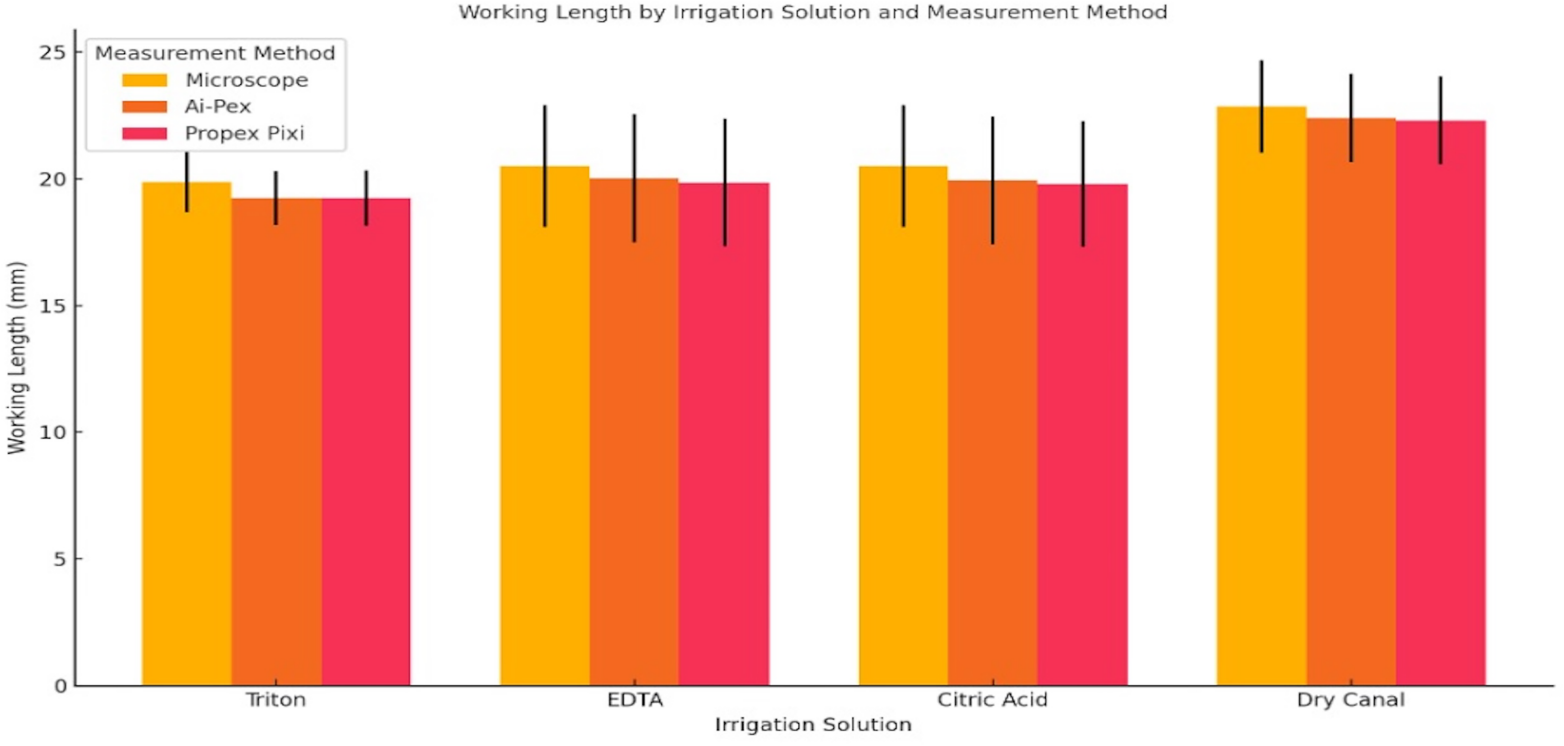
Comparison of WL by irrigant and apex locator (bar chart representation). Schematic illustration of the study workflow. Forty-four extracted teeth were randomly assigned to four irrigant groups (Triton, 17% EDTA, 10% citric acid, and dry canal), and measurements were obtained using two electronic apex locators (Ai-Pex and Propex Pixi). Statistical analysis included two-way ANOVA and *post hoc* tests.

Figure 4 illustrates the mean working length values obtained with different measurement methods under various irrigation conditions as a bar chart.

Accuracy analysis revealed that within a tolerance of ±0.5 mm, accuracy rates were 41.4% for Ai-Pex and 30% for Propex Pixi. When tolerance was extended to ±1.0 mm, accuracy increased to 87% and 80%, respectively, indicating clinically acceptable performance for both devices, with Ai-Pex showing slightly higher accuracy. These accuracy percentages were derived by calculating the proportion of measurements falling within ±0.5 mm and ±1.0 mm of the actual working length.

## Discussion

Accurate determination of WL is critical for successful root canal treatment, as both under- and over-instrumentation can negatively affect healing outcomes. EALs have become vital in modern endodontics due to their ability to provide consistent measurements with minimal radiation exposure (Abdelsalam & Hashem, 2020). However, the accuracy of these devices can be affected by the chemical composition and conductivity of the irrigants present in the canal during measurement.

In the present study, two multifrequency EALs (Ai-Pex and Propex Pixi) were evaluated under the influence of three irrigants: Triton, 17% EDTA, and 10% citric acid, as well as dry canal conditions. The results showed that all three irrigants produced statistically similar working length measurements across both EALs, while dry canals caused significant overestimation, regardless of the device used. These findings highlight the importance of keeping the canal moist during electronic length measurement and confirm that modern irrigants, including newly developed formulations, do not compromise the diagnostic accuracy of apex locators.

In this study, a 25× dental operating microscope was used to determine the actual working length instead of periapical radiographs, as it is reliable, especially in cases where the apical foramen opens laterally (Çelebi Keskin & Yalçın, 2025; Abdelsalam & Hashem, 2020; Diemer et al., 2022; Iparraguirre Nuñovero et al., 2021).

The accurate performance of the Ai-Pex device, which uses advanced digital signal processing (DSP) and multi-frequency impedance analysis, aligns with prior findings showing its reliability across different canal conditions (Çelebi Keskin & Yalçın, 2025; Baruah et al., 2018). While previous literature has supported the accuracy of Propex Pixi (Carvalho et al., 2010; Baldi et al., 2007), this study adds to the evidence by demonstrating that Ai-Pex provides equivalent performance, even when tested with a complex irrigant like Triton. This is particularly relevant, as limited data exist comparing Ai-Pex with established EALs under various irrigant conditions. Teja et al. (2025) reported that apex locator–integrated endomotors provide reliable measurements in the presence of irrigants, although minor deviations may occur depending on the irrigant type. Similarly, Kara and Subay demonstrated that Root ZX Mini, Raypex 6, and Propex Pixi maintained high accuracy within a ±0.5 mm range in both *in vivo* and *in vitro* conditions, with irrigant type or canal status not significantly compromising their performance (Kara & Sübay, 2025). These findings strengthen the clinical applicability of modern apex locators across diverse conditions. In the present study, the accuracy rates within ±0.5 mm were 41.4% for Ai-Pex and 30.0% for Propex Pixi, while accuracy within ±1.0 mm increased to 87.0% and 80.0%, respectively.

Although alginate does not perfectly imitate periapical tissues, it is one of the most frequently used materials for simulating *in vivo* conditions in apex locator studies. In this study, alginate was selected for its electroconductive properties, stable consistency, and ability to mimic the periodontal ligament, making it a dependable medium for electronic working length measurements (Baruah et al., 2018; Carvalho et al., 2010; Baldi et al., 2007).

One of the most distinctive features of this study is the inclusion of Triton, a novel all-in-one irrigant containing NaOCl, citric acid, and a combination of anionic surfactants. Unlike traditional protocols that require sequential application of NaOCl and chelating agents, Triton simplifies the irrigation regimen by offering simultaneous antimicrobial activity, smear layer removal, and organic tissue dissolution (Sheng et al., 2023). Despite its complex chemical profile, including anionic and nonionic surfactants such as sodium lauryl sulfate and ethoxylated alcohols, Triton did not interfere with apex locator function. This finding is particularly noteworthy considering that surfactants are known to alter electrical conductivity, potentially affecting EAL accuracy (Ozsezer, Inan & Aydin, 2007; Jain & Kapur, 2012).

The compatibility of Triton with EAL measurements may be attributed to its buffered pH environment and optimized ionic balance, which could minimize signal distortion during electronic measurement. This is consistent with the design of newer apex locators like Ai-Pex and Propex Pixi, which are engineered to perform accurately across a range of canal conditions by using multifrequency impedance analysis (Chukka et al., 2020; Shekarbaghani, Bolhari & Khalilak, 2024). Additionally, the stable readings observed in this study support the notion that modern EALs are less susceptible to chemical interference compared to earlier-generation devices (Berman & Hargreaves, 2020).

Findings from the dry canal group reinforce concerns raised in prior studies about overestimation of WL in the absence of conductive media (de Almeida Gardelin et al., 2023; Gürel, Kıvanç & Ekici, 2017). Although some literature has reported acceptable accuracy under dry conditions (Yahya, Alchalabi & Alkhalidi, 2024; Kang & Kim, 2008); these results may vary depending on the device type, measurement protocol, and apical morphology. In the present study, both EALs consistently overestimated WL by approximately 2 mm, suggesting that dry canal conditions should be avoided during electronic measurements, particularly when using multifrequency-based devices.

From a clinical perspective, the results support using combination irrigants like Triton during working length determination. Since Triton did not compromise the accuracy of either device tested, clinicians may consider adding it to their protocol to reduce treatment time and simplify irrigation without losing diagnostic reliability. To our knowledge, this is the first study to evaluate the performance of EALs in the presence of Triton. While Triton has been examined for its antimicrobial activity and smear layer removal capabilities (Bandoo et al., 2024; Castagnola et al., 2024), its effect on apex locator accuracy had not been previously assessed. This study thus provides new evidence supporting Triton’s clinical safety and compatibility with electronic length determination.

### Limitations and future perspectives

This study is the first to assess the performance of electronic apex locators in the presence of Triton, a novel all-in-one irrigant, and provides original evidence that its complex formulation does not compromise working length determination. While the *in vitro* design cannot fully replicate clinical variables such as apical resorption or canal curvature, and only two devices and single-rooted teeth were tested, these limitations do not diminish the relevance of the findings. Future studies should investigate different irrigant properties, additional apex locator models, and diverse tooth anatomies under clinical conditions. Further *in vivo* studies are warranted to validate the performance of Triton and electronic apex locators under real clinical conditions. Future studies may also focus on comparing Triton with other newly introduced endodontic irrigants to further strengthen the available evidence. Despite these limitations, the present results highlight the reliability of modern EALs even with advanced irrigant formulations like Triton.

### Clinical implications

Accurate determination of the working length remains one of the most critical steps in root canal therapy, as it directly influences treatment success and the long-term healing of periapical tissues. Modern electronic apex locators have significantly enhanced clinical efficiency by decreasing reliance on radiographs and minimizing radiation exposure. The present study offers the first evidence that Triton, a novel all-in-one irrigant with a complex chemical composition, does not compromise the accuracy of working length determination when utilized with contemporary electronic apex locators. This finding holds clinical significance, as Triton amalgamates the antimicrobial, chelating, and tissue-dissolving properties of traditional irrigants into a single solution, thereby potentially simplifying irrigation procedures and reducing chairside time. By demonstrating compatibility with electronic apex locator measurements, the results support the safe incorporation of Triton into clinical endodontic practice. Consequently, clinicians may consider employing its use not only as a time-efficient irrigant but also as a means of preserving diagnostic reliability during electronic working length assessment.

## Conclusion

This study showed that Triton, a novel all-in-one irrigant containing sodium hypochlorite, surfactants, and chelating agents, provided working length measurement accuracy comparable to EDTA and citric acid. Its compatibility with both Propex Pixi and Ai-Pex suggests safe integration with multifrequency EAL systems. By combining disinfection, tissue dissolution, and chelation in a single step, Triton represents a clinically viable option for simplifying irrigation protocols.

## Supplemental Information

10.7717/peerj.20872/supp-1 Supplemental Information 1 Raw data – Working length measurements. All working length measurements obtained with the microscope, Ai-Pex, and Propex Pixi under different irrigation conditions (Triton, NaOCl, EDTA, Citric acid, Dry canal).

10.7717/peerj.20872/supp-2 Supplemental Information 2 Raw data output – statistical processing. Processed mean values and calculated differences between measured and actual working lengths for all experimental groups.

## Additional Information and Declarations

### Data Availability

The following information was supplied regarding data availability:

The raw data used for statistical analysis are available in the Supplemental File.
